# A Case Study of Veteran Patient‐Researcher Partnerships in Mental Health Research and Practice: Three Recommendations From a Veteran Patient Engaged in Research (VPER)

**DOI:** 10.1111/hex.70508

**Published:** 2025-12-13

**Authors:** Madisen E. Brown, Hannah M. Bailey, Rachel P. Riendeau, Christopher J. Miller, Bo Kim, Eva N. Woodward, Colleen Turner

**Affiliations:** ^1^ VA Boston Healthcare System Center for Healthcare Organization and Implementation Research (CHOIR) Boston Massachusetts USA; ^2^ VA Center for Mental Healthcare and Outcomes Research Central Arkansas Veterans Healthcare System Little Rock Arkansas USA

**Keywords:** consumer engagement, partnership dynamics, patient engagement, patient‐centred outcomes research, Veteran consultant, Veteran research

## Abstract

**Introduction:**

Veteran patient partnerships in research improve patient‐centred healthcare outcomes within US Department of Veterans Affairs (VA) medical centres. To achieve this, researchers must contextualize perspectives, motivations, and contributions of Veteran patients engaged in research (VPERs) as valued consultants within a complex healthcare environment. Our objective was to investigate best practices for research teams partnering with VPERs by utilizing the expertise of our research team's own VPER. The parent project of this case study, Hybrid Controlled Trial to Implement Collaborative Care in General Mental Health, was approved by the VA Boston Healthcare System Institutional Review Board and deemed research.

**Objective:**

Provide three key recommendations when engaging military Veterans and/or VA Veteran Patients in research to facilitate sustained teamwork and integration.

**Study Design and Methodologies:**

This paper is structured as a qualitative descriptive data‐based case study. Two team members used a semi‐structured conversation guide to interview Dr. Colleen Turner, MSW, PhD, Lt. Col. (Ret., US Air Force Reserves) for one hour about her experience as a VPER on the 5‐year research project. This manuscript was then written collaboratively by members of the team, with heavy influence and editing from Colleen for details and accuracy.

**Results:**

Improving mental healthcare for Veterans motivated Colleen to serve as a VPER from 2015 to 2020. She used organizational and provider‐level mental health expertise gained during her Air Force service, applied her graduate social work training, and offered her experience as a VA patient. A diverse background and an ability to codeswitch helped her navigate the study and enriched the team's partnership dynamics. Through a qualitative interview with Colleen about her experience as a VPER, three recommendations emerged for research teams to better situate VPERs on studies: (1) ensure initial project literacy and provide ongoing support, (2) incorporate VPER goals into project work and (3) communicate both (a) offers of reasonable compensation and professional acknowledgement and (b) visible patient‐centred outcomes.

**Conclusion:**

This case study deepens the understanding of how to meaningfully incorporate VPERs into a partnered research study. Engagement starts early, continues throughout the study, and culminates with well‐communicated outcomes as they pertain to the researchers’ and VPERs’ goals. These recommendations closely align with widely accepted community‐engaged research practices and may guide ongoing and future studies to further improve patient engagement in research and the collaboration experiences of VPERs.

**Patient or Public Contribution:**

This manuscript focuses on Colleen's contributions to the design and conduct of a hybrid implementation/effectiveness study. Colleen also contributed to the manuscript itself. Uniquely, Dr. Turner brought her experience as a practitioner with master's and doctoral degrees in social work, as an institutional communications analyst, and as an independent author. Because of this, she was able to bring even more expertise to the project beyond just that of a patient, which is valuable in its own right. Colleen was involved in the research project from inception to conclusion, including study design, general analysis, and manuscript publication, with overall consultation throughout.

## 1 Introduction

Veterans possess a variety of valuable perspectives and lived experiences. These qualities bring depth and richness to the research world [1]. Traditionally, persons engaged in research in this context would be labelled ‘stakeholders’. Instead, we refer to them as Veteran patients engaged in research (VPERs), per the guidance of the Health Equity Style Guide released in 2020 by the Centers for Disease Control and Prevention [2]. VPERs are individuals who use US Department of Veterans Affairs (VA) resources as either Veterans or family members; they are not participants in the study itself but are instead compensated members of the research team. Researchers must contextualize Veteran patients’ viewpoints, motivations and contributions within a complex research environment if useful changes and sustainable outcomes in the healthcare system are to be achieved. While health systems researchers now pay more attention to the valuable role that VPERS and others with lived experiences may have on research teams, literature remains scarce, especially in marginalized and multifaceted populations such as US Veterans [3, 4, 5, 6]. This study seeks to add to the growing evidence‐based practice of engaging patients in research, particularly with research surrounding Veterans [7, 8].

## 2 Objective

Our objective was to provide recommendations for engaging military Veterans and/or VA Veteran Patients in research to facilitate sustained teamwork and integration.

## 3 Study Design and Methodologies

### 3.1 Study Context

The parent project of this case study, Hybrid Controlled Trial to Implement Collaborative Care in General Mental Health, was approved by the VA Boston Institutional Review Board (IRB) and deemed research. This case study is a qualitative data‐based paper, based primarily on VPER engagement and feedback. More details on the design of the parent trial can be found elsewhere [9, 10, 11]. Briefly, it was a hybrid implementation‐effectiveness trial that was intended to improve collaboration, teamwork, care coordination and clinical outcomes within outpatient general mental health teams.

### 3.2 The Role of the Veteran Patient Engaged in Research

In describing the role of the VPER in the project, we draw from Sheikhan and colleagues’ guidance for reporting on lived experience engagement [12]. In terms of initial engagement with the study team, Dr. Colleen Turner, MSW, PhD, Lt. Col. (Ret., US Air Force Reserves) had a longstanding working relationship serving as a consultant on prior projects. Thus, she was a natural fit to continue in that consultative role on the current project. The specific role involved providing both regular and ad hoc feedback on study design decisions and interpretations of findings during the duration of the study period (2015–2020). Regular feedback was obtained via attendance at quarterly meetings with the study team alongside other Veterans, family members, and stakeholders. Ad hoc feedback was obtained at irregular intervals, especially during the launch of the project, with emphasis on the best ways to structure high‐quality mental health care for Veterans. Colleen was paid as a member of the study team in a consultative role.

### 3.3 Interview Procedures

Two team members used a co‐designed semi‐structured interview guide to collect data from Colleen about her experiences as a VPER. This guide was collaboratively designed by the lead qualitative analyst and co‐investigator. Other team members reviewed the guide for clarity and relevance. The semi‐structured interview guide consisted of 7 questions and 11 follow‐up probes. The questions focused mainly on inquiring about a VPER's experience on a VA‐based research project. Two team members, both acting in coordinator roles, conducted the hour‐long interview with Colleen via telephone in 2021 using VA's secure national teleconferencing system. The interview was not recorded or transcribed due to time and budget constraints, but extensive notes were taken by the interviewers. These notes were saved in a team project file behind a VA firewall, in compliance with VA Boston IRB guidelines.

### 3.4 Analytic Plan

The research team collaboratively analysed the interview notes using emergent thematic coding and discussion. Identification of the themes was first conducted independently by each of the two interviewers, using their separate sets of notes from the interview. They compared their identified themes and discussed reaching preliminary consensus, before checking for consistency with Colleen to refine and finalize the identified themes. The team then collaboratively drafted a manuscript. Novelly, Colleen heavily influenced and edited this paper for details and accuracy. This method of co‐writing papers with VPERs and patients engaged in research is receiving growing support from the healthcare community, however, such articles remain scarce [13].

## 4 Results

### 4.1 Participant Background

This case study describes the values of one VPER and how those values shaped her expectations assisting the Hybrid Controlled Trial to Implement Collaborative Care in General Mental Health project. Foremost, improving mental healthcare for other Veterans motivated Dr. Colleen Turner, MSW, PhD, Lt. Col. (Ret., US Air Force Reserves) to serve as a VPER from 2015 to 2020 with the Behavioral Health Quality Enhancement Research Initiative (BH QUERI) Program, a team‐based mental healthcare implementation project [11].

Dr. Colleen Turner has been involved with VA in various roles over decades as a family advocate, intern, employee, volunteer and patient. She is a Vietnam‐era veteran with Air Force active duty, Reserve and National Guard experience. Her service spans 30 years, retiring as a Lieutenant Colonel, US Air Force Reserve. She holds an MSW and PhD from the School of Social Work at UCLA. After military service, she served 7 years as a Communication Analyst for the VA Office of Inspector General. In her time with the research team, she described drawing on experience from her many professional and experiential roles. Even with her diverse background, she sometimes felt ineffective in the research setting. The study's incremental processes seemed distant and removed from her mission to improve mental healthcare quality.

Colleen reported that she accrued mental health expertise at organizational and provider levels during military service and graduate education, but her VPER engagement officially began as a VA patient. She details much of her VA‐specific healthcare journey, between 1986 and 2011, through an executive summary titled, ‘One Veterans PTSD Journey’, which she authored herself and shared with a panel of national VA interdisciplinary providers [14]. In this paper she outlines problems within the system and proposes solutions. The short version: she experienced trouble with health assessments, accusations of malingering to access disability services, barriers to care, invalidated and disregarded pain and PTSD symptoms and experiences, a lack of educational tools about her care and options, a major culture gap between her health issues as a Veteran and physicians understanding them and difficulty navigating the disability claims process, just to name a few. While she makes important statements in her executive summary about the positive experiences she has had with VA care, it is the lengthy history of barriers and convoluted pathways to care that motivated her to become a VPER. She found it difficult to navigate the large healthcare system, the litany of appointments and referrals and make sense of the misaligned next steps for her diagnosis, and her concern for her fellow Veterans inspired her to action:

I kept thinking, “I'm strong enough to deal with this”, but wondered what must be happening to others as these processes could be horrific for them.

At her local VA hospital, Colleen formed a collegial friendship with a care provider, ‘Dr. A’. Dr. A encouraged her to write about her PTSD journey and later invited her to present her executive brief to clinicians. She went on to serve in an official capacity on VA councils with other VPERs. Memorably, in these meetings, Dr. A acknowledged Colleen's diverse perspectives by calling her ‘Dr. Turner’, ‘Colonel’ and or ‘Colleen’, which made her feel respected. In 2015, Dr. A recommended Colleen as a consultant for our study and BH QUERI. Colleen's engagement on this project significantly enhanced study methods and diversified team dynamics.

Reflecting on her time as a Veteran Patient Engaged in Research, Colleen emphasized how the research team ‘*really did have the right priorities and kept Veterans first*’. Despite frustration about larger VA change and slow site‐level progress, she felt the depth of the team's sincerity and shared her goal to improve VA mental healthcare. She also felt her involvement as a VPER reflected well on the research team's priorities. These realities motivated Colleen to continue contributing to VA research, even though she might not see immediate system‐level changes.

### 4.2 Lessons Learned

In discussing the findings from Colleen presented above, the author team derived three important recommendations (see Table 1) for research teams to consider when partnering with VPERs.

**Table 1:** Recommendations.

1	Ensure initial project literacy and provide ongoing support
2	Incorporate VPER goals
3	Communicate both (A) offers of reasonable compensation and professional acknowledgement and (B) visible patient‐centred outcomes

### 4.3 #1: Ensure Initial Project Literacy and Provide Ongoing Support

Colleen emphasized that VPERs need a comprehensive understanding of the research project and ongoing, supportive check‐ins throughout the study cycle to contribute deeply. While Colleen offered that she may be ‘*more informed than the average Veteran’*, she occasionally struggled to get a handle on the BH QUERI project. She shared that at the beginning of the project:

An organizational chart of all research team members, their roles, relationships, contact information, and the larger VA research context would've helped with my orientation.

Colleen also suggested improving VPERs’ introduction to the study by clarifying expectations and carving out opportunities for specific VPER contributions. Once Colleen understood the study more clearly, she was better able to further the goals of the research.

Colleen joined the study when the funding began, a time which marks an official study start date, but the research team already had years of pre‐funded project preparations under their belts. Consequently, Colleen felt as though she was arriving late.

I wasn't involved with the study pilot or grant development, so I felt left behind due to joining after those initial phases. I was uncomfortable asking questions related to issues I knew they would think I already understood. I needed to be able to state what they were doing, and how, in my own words, but no one asked, and I didn't offer.

This feedback helped the team understand just how much VPERs can benefit from summaries of projects written in lay language.

Colleen's suggestions demonstrate two procedural and relational lessons: (1) the team must create a strong initial onboarding process, which may include working with them to set a research agenda and working to minimize team hierarchies [7] and instill individual value; and (2) the team must support VPERs understanding of study goals and methods throughout the project's entirety.

### 4.4 #2: Incorporate VPER Goals

Colleen emphasized the importance of incorporating the VPERs’ own goals by mutually setting aims and expectations regarding the scope of the project. Colleen's expectations about her impact on, and goals for, the project differed from the expectations and strategies of the research team. This was due, in part, to poor communication from the team regarding her direct contributions to research. Colleen originally thought she ‘*would be making a big difference*’ as a VPER but classified the changes she influenced as ‘*very small’*.

Colleen's background enabled her to codeswitch among intersecting military, provider, and patient perspectives [15]. Blanchard [16] define codeswitching as ‘shifting from the linguistic system of one language or dialect to that of another’. This is a foundational definition which helps researchers move one step further into a sociolinguistic understanding of how VPERs operate within a team. A more nuanced and particularly relevant explanation of codeswitching is ‘selection or alteration of language elements to match the context of the interaction: this practice may include linguistic and extra‐linguistic elements like identity, norms, culture, etc.’ [17]. An example of codeswitching in a research setting could be moving between healthcare language within the VA, which is laden with acronyms and research terms, to write a grant, to more lay and discussion‐based language about the experience of a healthcare visit and interaction with a provider. For Colleen, codeswitching helped her better understand the project researchers’ perspectives, the stakeholders involved and the scientific aims within the VA landscape. It also empowered her to communicate the perspective of the Veteran patient to a team of scientists and academics researchers who can be disconnected from the clinical processes and interactions.

Colleen reflected on this skill's value, saying she often felt she was acting as:

…a translator[,] has been the most important aspect of my contributions, to be able to speak the “dialects” of Veterans, patients, administrators, researchers, and mental health providers.

However, even this experience as a go‐between left her unprepared for the sharp contrast between the changes she championed within VA mental healthcare and research's incremental interventions. Colleen felt as though she and the team were not making ground‐level impacts during the project. While the intervention was not rolled out to her medical centre until later down the road, Colleen explained:

I kept hearing about BHIP at my VA, but I felt unable to constructively influence it.

This unfelt change in clinical care delivery at her VA led her to lose her ‘*naive mentality about how the research would have an influence on everyday VA activities’.* When the study results were published, she had ‘*mixed feelings’* about the significance of the main outcomes. Colleen concluded that the research yielded ‘*a result you can feel very proud about’* but still felt researchers ‘*could do so much more’* if they focused directly on problems identified by Veterans. From this contribution, the research team learned the importance of co‐creating goals with VPERs that fit the study trajectory and aims but also incorporating the VPER's own goals and ideas.

### 4.5 #3: Communicate Both (A) Offers of Reasonable Compensation and Professional Acknowledgement and (B) Visible Patient‐Centred Outcomes

Colleen noted that reasonable compensation and professional, personalized acknowledgements of impact are important ways to show VPERs their value to a research team and project, but communicating visible healthcare changes that improve patient‐centred outcomes aligned with VPERs’ motivations for collaborating on research matters most. Overall, Colleen felt personally valued by the VA Boston research team, even as its goals were secondary to her motivation to improve mental healthcare for Veterans. She also appreciated receiving compensation for her time and efforts on the study. She largely measured her value to the research by how she saw mental healthcare improve, though she struggled to pinpoint this change. The interviewers learned that the research team had not adequately communicated to Colleen the successes of her suggestions. For example, she was surprised to learn that the project's implementation manual, distributed to VA mental health clinics nationally, cited her directly.

Despite gaps in communication, Colleen appreciated her working relationship with the team's investigators and other colleagues. ‘*During the meetings, I was never left out, and if I didn't participate, I was drawn in’,* she noted, recalling how the team made continuous efforts to include her. This active inclusion of her opinions made her feel ‘*extraordinarily respected by all of the team members’.*

At the end of the study, the research team presented Colleen with a personalized certificate of gratitude, which she felt conveyed thoughtfulness, admiration and sincerity from the team. She plans to display this in her home/office. From this contribution arose three additional lessons: (1) when possible, appropriately compensating VPERs for their time strongly signals value; (2) researchers must specifically record VPER contributions and intentionally communicate them back to the VPER, especially when ground‐level research impacts may be less visible at the patient level; and (3) official and personalized forms of appreciation can be very meaningful.

## 5 Discussion

In this case study, we describe the experiences and lessons learned from one Veteran Patient Engaged in Research, Colleen. Results from this case study suggest that researchers cannot rely on the initial explanation and descriptions of a project to answer all of a VPERs questions throughout the entirety of the project. A paper description and a kick off meeting are good starting points for orientation, but as the research project operates in real time, consistent check‐ins about project understanding, literacy, and practice, are pivotal. It's important to make sure the VPER does not feel left behind as the project progresses through its timeline and phases. Our team also learned just how valuable it is to work with someone who has in‐depth experience in the healthcare system that we are targeting for research and/or implementation. No amount of classroom case studies or graduate theses can replace or mirror the lived experience of a patient. The perspective VPERs bring to the table is irreplaceable in this way.

Three specific recommendations for engaging stakeholders in similar research projects emerged from this case study, including (1) ensuring initial and ongoing project literacy, (2) incorporating VPER goals into the working relationship with the study team and (3) communicating both (A) reasonable compensation and professional acknowledgement and (B) visible patient‐centred outcomes. In broad strokes, these are consistent with previous work related to patient engagement in research [4, 5, 6,12, 13].

Based on our findings, we concluded that it was important to acknowledge the variety of identities carried by Colleen, such as that of a military Veteran, patient and doctoral‐level provider. Attending to only one of these roles, rather than codeswitching to draw on the breadth of Colleen's experiences, would have risked limiting her impact on the project. An unexpected takeaway for the team was how Colleen helped us learn to codeswitch in real time to close the gap between “medical speak” and “public speak”. As operators within the VA healthcare system, we have blind spots that can impact our communication of scientific information to patients seeking care. By using her relevant backgrounds and experiences, Colleen played a crucial role in helping the team communicate complex medical information into accessible patient‐centred language, which helps bridge the gap between research teams and Veteran patients [18].

## 6 Limitations

It is important to note that these insights and conclusions are individual and not necessarily generalizable. The VPER featured in the study has a unique background, including master's and doctoral degrees in social work, extended time in the military, and service in a federal workplace. Taken together, these are not typical of most VPERs. These extensive credentials made Colleen uniquely appropriate for the BH QUERI project, but it is important to note that not all VPERs need to have such extensive attributes to meaningfully contribute. Often, having served in the military and being a VA patient are the most important qualifying characteristics for engaging in VA research.

## 7 Conclusion

When asked if she would recommend other Veterans to engage in VA research, Colleen's immediate, unequivocal answer was, ‘*Absolutely!*’. While Colleen was especially enabled to engage in research because of her background, all Veterans have myriad lived experiences that may contribute to research. This fact, coupled with Colleen's story, should encourage researchers to actively seek Veteran perspectives in their work to reinforce the validity and power of those Veterans' experiences.

Colleen has heard important perspectives from other Veterans that need to be disseminated throughout VA and the research community more broadly, especially regarding mental healthcare. The research team will incorporate Colleen's contributions and the important lessons from this study (e.g., ensuring project literacy, integrating VPER goals and communicating visible outcomes) into ongoing and future projects to further improve the collaboration experiences of VPERs and the research teams with whom they consult. See Table 2 for a list of abbreviations used throughout the paper.

**Table 2:** Acronyms.

BH QUERI	Behavioral Health Quality Enhancement Research Initiative
IRB	Institutional Review Board
PTSD	Post traumatic stress disorder
VA	(Department of) Veterans Affairs
VPER	Veteran patients engaged in research

## Author Contributions

**Madisen E. Brown:** conceptualization (equal), data curation (equal), formal analysis (equal), methodology (equal), project administration (lead), visualization (lead), writing – original draft preparation (lead), writing – review and editing (lead). **Hannah M. Bailey:** writing – review and editing (equal). **Rachel P. Riendeau:** conceptualization (equal), methodology (equal), data curation (equal), writing – review and editing (equal). **Christopher J. Miller:** supervision (lead), funding acquisition (lead), writing – review and analysis (equal), conceptualizatoin (equal), methodology (equal). **Bo Kim:** conceptualization (equal), methodology (equal), writing – review and editing (equal). **Eva N. Woodward:** writing – review and editing (equal). **Colleen Turner:** writing – review and editing (equal), conceptualization (equal).

## Ethics Statement

The authors have nothing to report.

## Consent

The study was conducted independently of any one project, with the consent of the Veteran consultant.

## Conflicts of Interest

The authors declare no conflicts of interest.

## Clinical Trial Registration

The authors have nothing to report.

## Data Availability

The authors have nothing to report.
